# Postoperative fluid collection at the proximal neurovascular bundle level is associated with early pain and reduced knee extensor strength after direct anterior total hip arthroplasty

**DOI:** 10.1186/s42836-026-00443-1

**Published:** 2026-11-09

**Authors:** Haruki Osanami, Yuki Kitta, Akira Inoue, Takatoshi Kaneko, Yuki Hirai, Koji Ugai, Eiki Tsushima

**Affiliations:** 1 Department of Rehabilitation, Keiyu Orthopaedic Hospital, Tatebayashi, Japan; 2 Department of Orthopaedic Surgery, Keiyu Orthopaedic Hospital, Tatebayashi, Japan; 3 Graduate School of Health Sciences, Hirosaki University, Hirosaki, Aomori, Japan

**Keywords:** Total hip arthroplasty, Direct anterior approach, Motor nerve to the vastus lateralis, Lateral femoral circumflex artery, Postoperative fluid collection, Ultrasound

## Abstract

**Background:**

Early anterolateral thigh pain after direct anterior total hip arthroplasty (DAA-THA) is common, and more severe symptoms than expected in the usual postoperative course can be difficult to interpret clinically. This study evaluated whether a landmark-based ultrasonographic pattern of postoperative fluid collection (POFC) at a reference level corresponding to a reported course of the proximal neurovascular bundle is associated with early pain and knee extensor strength.

**Methods:**

In this prospective cohort study, ultrasonography was performed at 1 week postoperatively in 182 patients (182 hips) who underwent DAA-THA. At a prespecified reference cross-section corresponding to a reported level where the proximal neurovascular bundle crosses the femoral midline, POFC extending medially beyond the femoral midline was classified as a midline-crossing POFC. Co-primary outcomes were proximal anterolateral thigh pain while walking, assessed using a numerical rating scale, and knee extension torque at 1 week. *P*-values for the co-primary outcomes were Holm-adjusted. The Western Ontario and McMaster Universities Osteoarthritis Index (WOMAC) subscales were analyzed longitudinally.

**Results:**

Of the 182 hips, 37 were classified as midline-crossing POFC and 145 as non–midline-crossing POFC. Compared with the non–midline-crossing group, the midline-crossing group had greater proximal anterolateral thigh pain during walking (adjusted mean difference, 3.35 points; 95% confidence interval [CI], 2.88–3.81; Holm-adjusted *p* < 0.001) and lower knee extension torque (adjusted mean difference, − 0.31 Nm/kg; 95% CI, − 0.38 to − 0.24; Holm-adjusted *p* < 0.001). WOMAC pain scores showed a significant group-by-time interaction.

**Conclusions:**

Midline-crossing POFC at 1 week after DAA-THA was associated with greater early walking pain and lower knee extensor strength. Ultrasound assessment at this reference level may provide useful adjunctive clinical information for selected patients whose early postoperative anterolateral thigh pain and reduced knee extensor performance appear greater than expected.

**Supplementary Information:**

The online version contains supplementary material available at https://doi.org/10.1186/s42836-026-00443-1.

## Introduction

A direct anterior approach (DAA) for total hip arthroplasty (THA) offers advantages, such as reduced early postoperative pain and faster functional recovery [1–3]. Recent network meta-analyses have further characterized DAA-related outcomes. Comparisons of DAA with SuperPATH and posterior approaches have shown broadly similar short-term outcomes for many measures, although differences in selected perioperative outcomes, including intraoperative blood loss, have been reported [4]. In addition, within DAA, the use of a traction table may influence selected perioperative outcomes, such as blood loss and periprosthetic fracture risk, whereas most surgical, radiological, functional, and other complication outcomes appear comparable between traction-table and standard-table techniques [5]. Nevertheless, thigh pain has been reported after DAA-THA [6]. Experimental pain studies have shown that pain involving the quadriceps or knee can reduce quadriceps activation and knee extension torque and alter knee extensor function during walking [7, 8]. One possible contributing factor to early anterolateral thigh symptoms is the neurovascular structure adjacent to the distal part of the DAA interval.

Distal to the DAA interval lies a proximal neurovascular bundle comprising the proximal main branch of the motor nerve to the vastus lateralis (MNVL) and transverse branch of the lateral femoral circumflex artery (LFCA) [9, 10]. Given the proximity of this neurovascular bundle to the distal DAA interval, postoperative changes in the local tissue environment around this region could plausibly be associated with anterolateral thigh pain. Because the MNVL provides motor innervation to the vastus lateralis, this anatomical relationship also provides a plausible basis for an association with knee extensor performance, although these anatomical considerations do not establish direct causal relationships. Anatomical studies have suggested that intraoperative manipulation can affect this region [10–12]; however, its relationship with postoperative findings has not been sufficiently investigated.

Postoperative fluid collection (POFC) may occur after THA [13]. Hematoma and seroma are representative types of POFC [14]. Hematoma may also be observed in the periarticular soft tissues or thigh during the early postoperative period after THA [15]. Although POFCs are often asymptomatic or transient, case reports have described postoperative neurologic symptoms associated with hematoma or seroma located adjacent to neural structures after THA [16–21]. DAA-specific evidence regarding hematoma-related compressive neuropathy remains limited; however, iliopsoas hematoma after THA performed through a minimally invasive modified direct anterior approach has been reported, with the patient presenting with acute pain and loss of strength in the affected limb [16]. In addition, hematoma and seroma have been reported as wound complications after DAA-THA [22], and a review of nerve injuries after DAA-THA identified hematoma-related compression after anticoagulation as a potential cause of femoral nerve palsy [23]. These observations suggest that the anatomical location of a POFC, rather than its presence alone, may be clinically relevant. However, no in vivo study has evaluated whether the positional relationship between POFC and the estimated course of the proximal neurovascular bundle is associated with early postoperative symptoms after DAA-THA.

Ultrasound (US) is useful in evaluating POFC after THA [24, 25] and visualizing neurovascular structures [26]. Therefore, we defined a reference cross-section at the level where the proximal neurovascular bundle crosses the femoral midline and examined whether POFC extending medially beyond the femoral midline on this cross-section was associated with anterolateral thigh pain and knee extension strength. We hypothesized that a midline-crossing POFC is associated with greater walking pain and lower knee extension strength at 1 week postoperatively.

## Materials and methods

### Study design and participants

This single-center prospective cohort study was conducted in accordance with the Declaration of Helsinki and approved by the institutional review boards of the participating institutions (approval numbers: 3711 and T2025-008). All participants provided written informed consent.

**Fig. 1:**
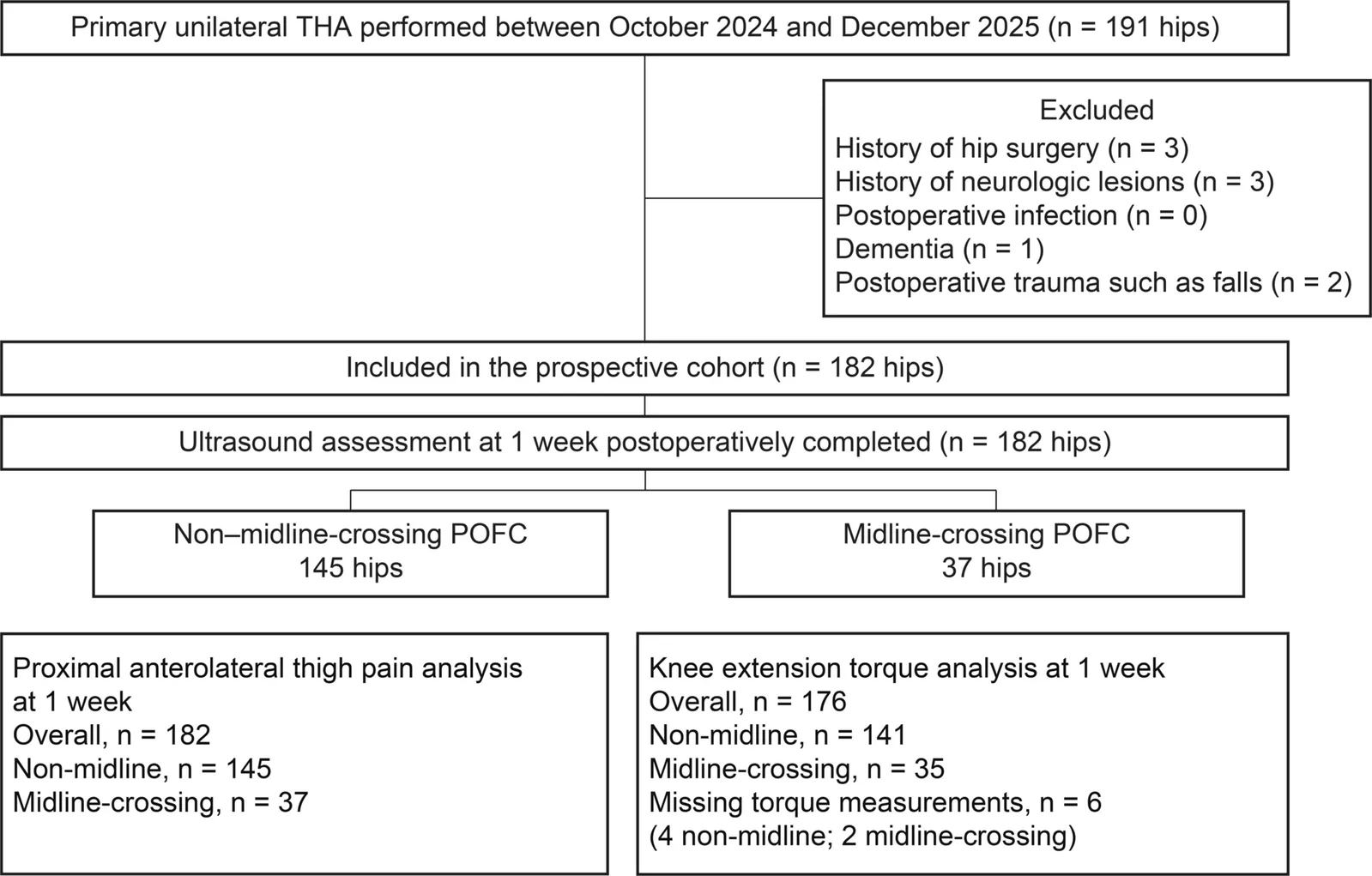
Participants flowchart and population analysis. THA, total hip arthroplasty; POFC, postoperative fluid collection

This study included 191 consecutive patients (191 hips) who underwent primary unilateral THA between October 2024 and December 2025. Patients with a history of hip surgery, neurological lesions, postoperative infection, complications caused by postoperative trauma, such as falls, and dementia were excluded. After exclusion of 9 patients (9 hips), 182 were finally included (182 hips; age, 67.4 ± 9.9 years; men, 27 and women, 155; and body mass index [BMI], 25.1 ± 4.3 kg/m^2^) (Fig. 1). According to the Tönnis classification system [27], 23 hips were grade 2, and 159 were grade 3.

### Surgical technique and perioperative management

All procedures were performed by a single experienced surgeon under general anesthesia, with patients in the supine position using the DAA. The skin incision was approximately 10 cm and was adjusted according to deformity and body habitus. A short stem was used in all patients. Hemostasis was achieved intraoperatively and before wound closure using electrocautery. Postoperative drains were not used as part of the standardized institutional perioperative protocol, which had been established before initiation of the present study and was applied consistently to all participants. As part of the blood management protocol, tranexamic acid 2 g was administered intravenously once during surgery. In patients receiving preoperative antiplatelet or anticoagulant therapy, perioperative discontinuation and resumption were managed according to a standardized institutional protocol in consultation with the prescribing physician. Antiplatelet agents were generally discontinued 7 days before surgery, and anticoagulants were withheld for 1–2 days preoperatively according to their pharmacologic characteristics. These medications were generally resumed on postoperative day 2 after confirmation of hemostasis and the clinical condition.

Perioperative pain management was also standardized according to the institutional protocol. A pericapsular nerve group (PENG) block was performed immediately before surgery, and fentanyl-based intravenous patient-controlled analgesia was used until postoperative day 1. After discontinuation of intravenous patient-controlled analgesia, oral analgesia consisted of loxoprofen sodium 60 mg three times daily. Acetaminophen 500 mg three times daily was used as a predefined alternative in patients with contraindications to non-steroidal anti-inflammatory drugs. No periarticular local infiltration analgesia was administered.

Postoperative rehabilitation was initiated on postoperative day 1 and continued until hospital discharge. Physical therapy was generally provided once daily for approximately 40 min per session and included gait training, hip range-of-motion exercises, and strengthening exercises. The duration and distance of gait training were not fixed but were individualized by the treating physical therapist according to each patient’s functional ability and clinical status; gait-specific duration and distance were not systematically recorded. Gait training was initiated with a walker and subsequently advanced to cane-assisted or independent ambulation according to each patient’s functional ability. For a post hoc descriptive assessment, walking independence was defined as the ability to ambulate at least 50 m without physical assistance, either with a cane or without an assistive device, as determined by the treating physical therapist. The first postoperative day on which this criterion was documented was abstracted from physical therapy records without knowledge of the POFC classification. Because physical therapy was not routinely provided on Sundays, this measure represents the first documented rather than necessarily the exact day of achievement. Walking without an assistive device was not required for discharge; from a walking standpoint, safe ambulation with a cane or without an assistive device was considered sufficient. Postoperative length of stay was defined as the number of days from surgery to hospital discharge. At our institution, postoperative hospitalization after DAA-THA was generally planned for approximately 14 days as part of the standard clinical pathway. Ice application was used as part of routine postoperative care from postoperative day 1 until hospital discharge. It was applied around the surgical wound for approximately 20 min per session, generally up to twice daily, and its use was not determined by pain severity, swelling, or POFC findings.

### US examination

US examination was performed primarily in B-mode using a SONIMAGE HS1 (Konica Minolta, Inc., Japan) with an 18-MHz high-frequency linear probe. Participants were examined in the supine position with hips in neutral alignment, and imaging parameters (depth, focus, gain, and probe pressure) were standardized. Based on prior anatomical studies [9, 10, 12], the proximal neurovascular bundle was identified using the proximal main branch of the motor nerve to the vastus lateralis (MNVL) and the transverse branch of the lateral femoral circumflex artery (LFCA). The MNVL appeared as a hyperechoic linear structure extending into the vastus lateralis, while the LFCA was identified using Doppler findings and arterial pulsation [26].

**Fig. 2:**
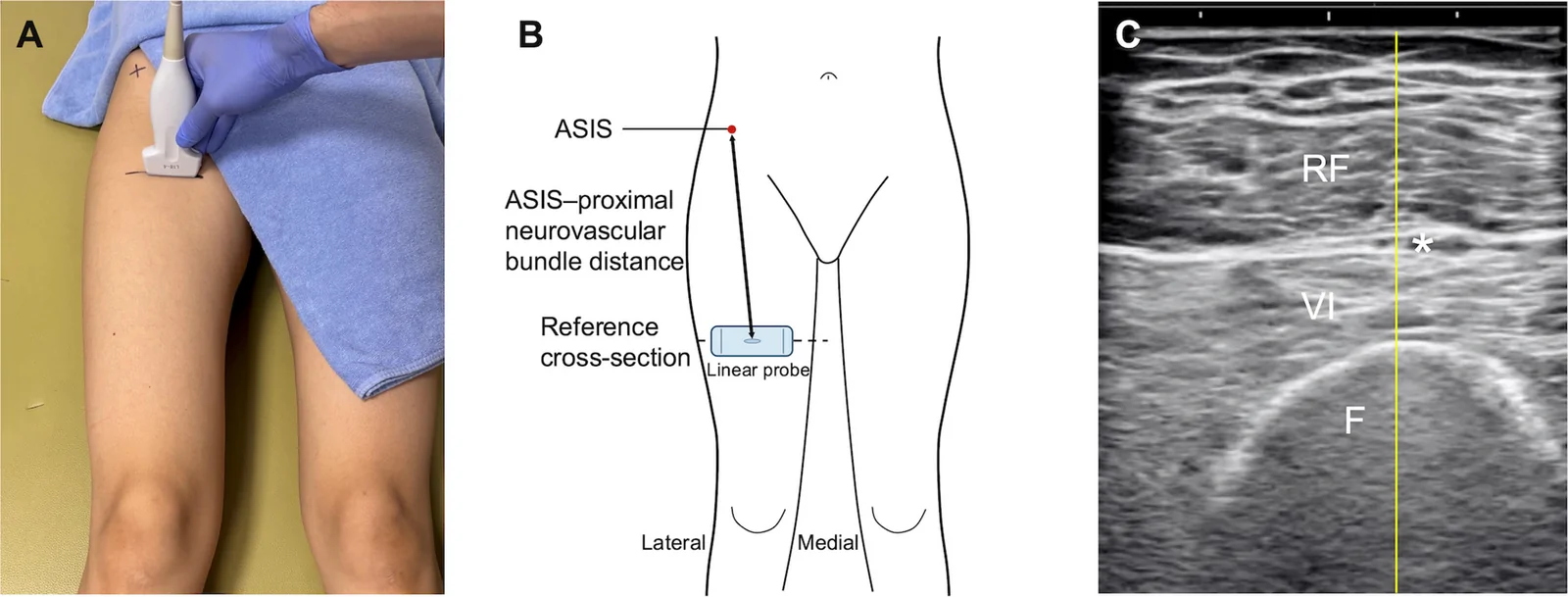
Ultrasonographic localization of the proximal neurovascular bundle and reference cross-section. **A** Representative photograph showing placement of the linear ultrasound probe at the reference cross-section. **B** Schematic diagram illustrating the body-surface landmarks and markings used to identify and reproduce the reference cross-section, including the anterior superior iliac spine (ASIS), the reference cross-section, and the ASIS–proximal neurovascular bundle distance used for postoperative reproduction of the cross-section. **C** Representative preoperative ultrasonographic image at the reference cross-section in a 77-year-old woman. *Proximal neurovascular bundle comprising the proximal main branch of the motor nerve to the vastus lateralis muscle (MNVL) and the transverse branch of the lateral femoral circumflex artery (LFCA). Yellow line, femoral midline, defined as the line passing through the midpoint between the medial and lateral femoral cortices on the short-axis image; RF, rectus femoris; VI, vastus intermedius; F, femoral bone

Preoperatively, following the study by Ghijselings et al. [10], a transverse image at the level where the proximal neurovascular bundle crosses the femoral midline was defined as the reference cross-section (Fig. 2). The femoral midline was defined as the midpoint between the medial and lateral cortices on short-axis imaging. This level was marked on the skin, and the distance from this level to the anterior superior iliac spine (ASIS) was recorded as the ASIS–proximal neurovascular bundle distance.

**Fig. 3:**
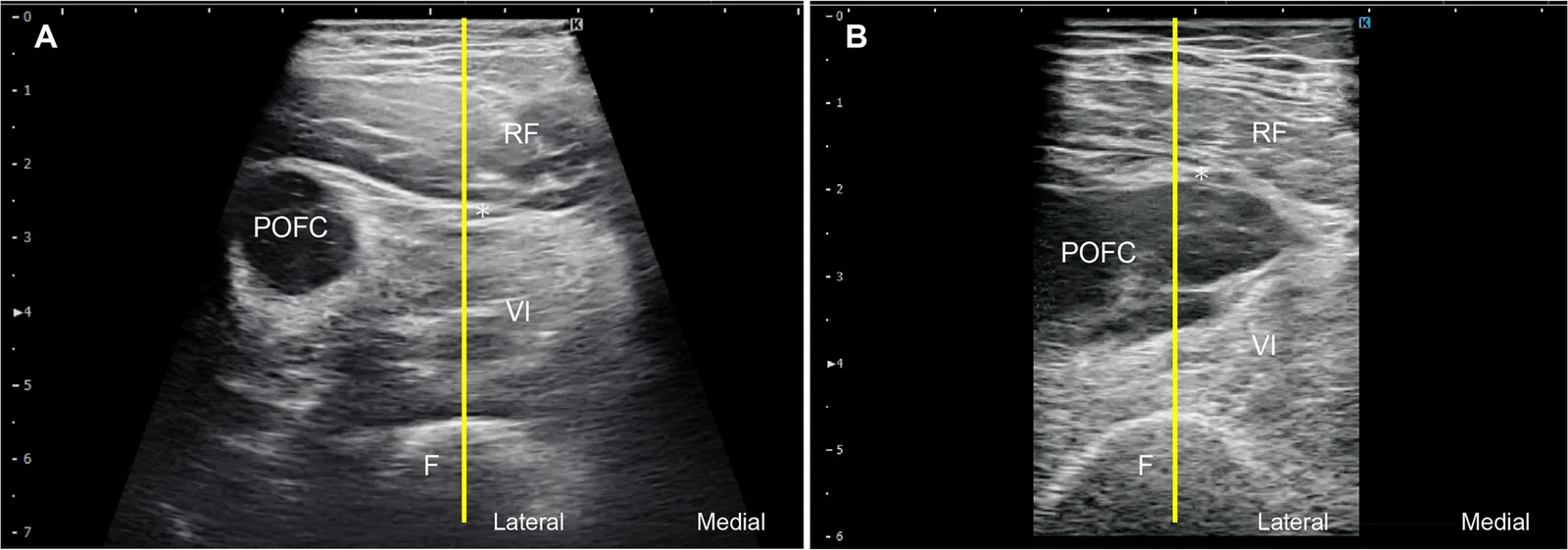
Landmark-based classification of POFC at the reference cross-section after DAA-THA. **A** Non–midline-crossing POFC group: Ultrasonographic findings at 1 week postoperatively in a 75-year-old woman. The POFC does not extend medially beyond the femoral midline, which served as the prespecified positional landmark for the classification. **B** Midline-crossing POFC group: Ultrasonographic findings at 1 week postoperatively in the same 77-year-old woman shown in Fig. 2C. The POFC extends medially beyond the femoral midline, which served as the prespecified positional landmark for the classification. *Estimated location of the proximal neurovascular bundle identified preoperatively. POFC, postoperative fluid collection; yellow line, femoral midline; RF, rectus femoris; VI, vastus intermedius; F, femoral bone; DAA, direct anterior approach; THA, total hip arthroplasty

At 1 week postoperatively, the same cross-section was reproduced using preoperative markings and the ASIS–proximal neurovascular bundle distance. The 1-week postoperative time point was selected a priori for ultrasound assessment to detect early POFC, because hematomas typically become apparent within 48–72 h and seromas usually develop within 2 weeks after surgery or trauma [14]. To preserve measurement consistency across patients, analyses were restricted to the prespecified reference cross-section. POFC was defined as a well-demarcated hypoechoic or mixed-echogenic fluid collection without internal Doppler flow [25, 28, 29]. Because the reference cross-section was defined preoperatively at the level where the proximal neurovascular bundle crosses the femoral midline, medial extension of POFC beyond the femoral midline was prespecified as the positional criterion for the midline-crossing classification. POFC extending medially beyond the femoral midline was classified as midline-crossing POFC, whereas POFC not extending beyond it was classified as non–midline-crossing POFC (Fig. 3). This anatomy-based criterion was not derived from the clinical outcomes and was intended as a reproducible operational definition rather than as a biologically validated threshold or as evidence of direct neurovascular contact or compression. In patients enrolled after longitudinal ultrasound follow-up was added to the protocol, the midline-crossing POFC pattern was also assessed at 2 and 6 weeks to describe its temporal course.

**Fig. 4:**
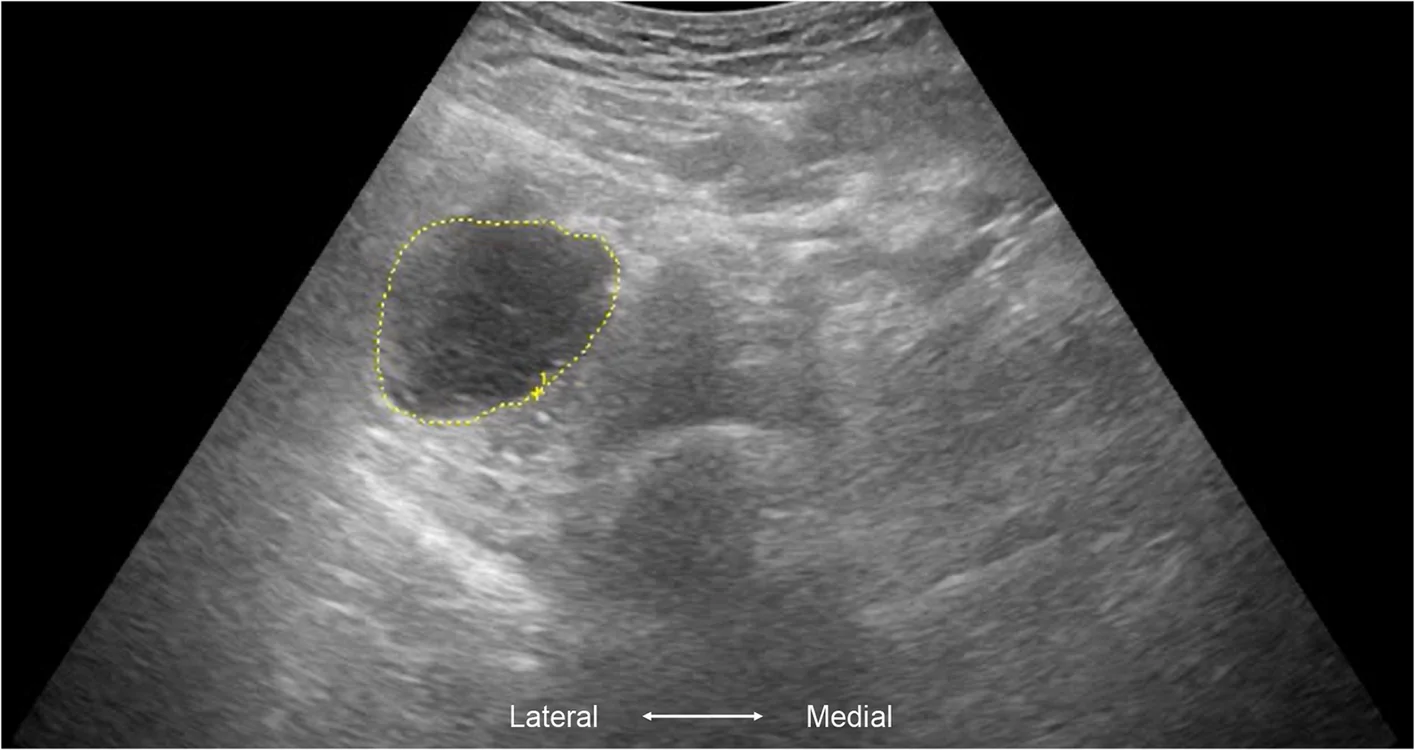
Measurement of the POFC area after DAA-THA. POFC, postoperative fluid collection; DAA, direct anterior approach; THA, total hip arthroplasty

POFC area was measured at 1 week as a two-dimensional area on the reference plane [30, 31] using a LOGIQ S8 system (GE Healthcare). The margin was manually traced, and the mean of two measurements was analyzed (Fig. 4).

### Blinding and reproducibility

Ultrasound examinations in the main study were performed by one primary examiner, who was not involved in the assessment of pain or muscle strength. Interpretation of ultrasound findings and group classification were performed with blinding to clinical outcomes, including pain, muscle strength, and clinical course information. Agreement for the assessment of midline-crossing POFC was evaluated using Cohen’s kappa coefficient based on anonymized ultrasound image sets from all 182 patients. Intra-rater agreement was assessed by repeated classification performed by the same primary examiner at an interval of at least 1 week; the results of the first assessment were unavailable during the second assessment. Inter-rater agreement was assessed by independent classification of the same anonymized image sets by the primary examiner and a second physical therapist with more than 5 years of musculoskeletal ultrasound experience.

Intra- and inter-rater reliabilities of the ASIS–proximal neurovascular bundle distance and POFC area were assessed in 20 patients (20 hips) from the study cohort. Intra-rater reliability was assessed by repeated measurements performed by the same primary examiner at an interval of 1 day. Inter-rater reliability was assessed by independent measurements performed by the primary examiner and the second physical therapist. To standardize the measurement procedures, the assessors completed a 1-month training period before reliability testing, consisting of at least three 90-min training sessions per week.

### Outcome measures

The co-primary outcomes were proximal anterolateral thigh pain during walking, assessed using the numerical rating scale (NRS), and knee extension torque (Nm/kg), both measured at 1 week postoperatively. The 1-week time point was selected a priori as the primary assessment time point because it was considered clinically relevant for evaluating early postoperative pain and lower-extremity muscle dysfunction during the early recovery phase after THA [32]. Pain location was recorded on a body chart [33] and evaluated separately for the proximal (co-primary) and distal (secondary) anterolateral thigh. Pain during walking was assessed immediately after a standardized 10-m walk at a self-selected pace using the patient’s usual assistive device. To minimize variability, assessments were conducted 2–4 h after analgesic administration. Knee extensor strength on the operated side was measured as maximal isometric contraction using a handheld dynamometer (Mobie MT-100, Sakai Medical). The participants were seated with their hips and knees flexed to 90°, and the device was positioned at the distal anterior lower leg [34, 35]. Measurements were obtained preoperatively and at 1 week, then adjusted for lower leg length and body weight to calculate torque (Nm/kg) [36].

Secondary outcomes included the Western Ontario and McMaster Universities Osteoarthritis Index (WOMAC), thigh circumference change, sensory disturbance, and blood loss. WOMAC was assessed preoperatively and at 1, 2, and 6 weeks [37]. At each postoperative assessment, participants were instructed to rate their condition at the respective assessment time point. Each WOMAC item was scored on a 5-point scale from 0 to 4, with higher scores indicating greater symptom severity or functional difficulty. The possible subscale scores ranged from 0 to 20 for pain, 0 to 8 for stiffness, and 0 to 68 for physical function. Thigh circumference was measured on the operated side, with the patient in the supine position, at the reference level used for the ultrasonographic assessment, where the proximal neurovascular bundle was identified preoperatively as crossing the femoral midline. The same measurement level was reproduced at 1 week postoperatively using the preoperatively recorded ASIS–proximal neurovascular bundle distance. The change in thigh circumference from preoperatively to 1 week postoperatively was used for analysis [38, 39]. Sensory disturbance was recorded by interview and light-touch comparison. Blood loss was assessed as total blood loss and hidden blood loss (HBL) using previously reported formulas based on perioperative hemoglobin and hematocrit values [40–42].

### Data analyses

An a priori sample size calculation was performed using G*Power version 3.1 (Heinrich Heine University Düsseldorf, Düsseldorf, Germany). Because no previous study had evaluated this landmark-based POFC classification after DAA-THA, a medium effect size was assumed (Cohen’s d = 0.50). For a two-sided independent-samples t-test with an α level of 0.05, 80% power, and an allocation ratio of 2.17 based on pilot data, the required sample size was estimated to be 47 patients in the smaller group and 101 patients in the larger group, for a total of 148 patients. The final cohort included 182 patients. Because group allocation in this prospective cohort study was determined by the observed postoperative POFC classification, the precision of the estimates was interpreted using adjusted effect estimates and 95% confidence intervals.

Statistical analyses were performed using R Commander version 4.0.2. Reliability of continuous measures was assessed using intra-class correlation coefficients (ICC), with ICC (1,1) for intra-rater reliability and ICC (2,1) for inter-rater reliability. Agreement for the assessment of the midline-crossing POFC was evaluated using Cohen’s kappa coefficient with 95% confidence intervals (CIs) and percentage agreement. Minimal detectable difference at 95% confidence level (MDD95%) was calculated using a previously reported formula [43]. Baseline characteristics and secondary outcomes were compared using the Student t-test or Mann–Whitney U test for continuous variables and Fisher’s exact test for categorical variables, as appropriate. Covariates were selected a priori based on their clinical plausibility and potential association with postoperative pain and muscle strength.

For the co-primary outcomes, an analysis of covariance (ANCOVA) was used to estimate adjusted mean differences, 95% CIs, and *p*-values. The main model included age, sex, BMI, Tönnis classification, and operative time. Additionally, preoperative knee extension torque was included in the knee extension torque outcome. Proximal NRS analysis included the 182 hips. For knee extension torque, 6 of the 182 hips without either preoperative or postoperative measurements were excluded, leaving 176 hips for complete case analysis. Missing torque data occurred in 4 of 145 hips in the non–midline-crossing POFC group and in 2 of 37 hips in the midline-crossing POFC group. All missing measurements were due to procedural reasons rather than an inability to perform testing because of pain or other symptoms. Homogeneity of regression slopes was confirmed, and Holm adjustment was applied to the co-primary outcomes. Distal anterolateral thigh pain during walking was analyzed using the same approach as the secondary exploratory outcome. A post hoc descriptive overlap analysis was performed among patients with complete data for both co-primary outcomes. Greater walking pain was defined as proximal anterolateral thigh pain during walking in the highest quartile, and knee weakness was defined as knee extension torque at 1 week in the lowest quartile; the overlap was summarized using counts and percentages without additional hypothesis testing.

Prespecified sensitivity analyses were additionally adjusted for the POFC area, HBL, antithrombotic drug use, and anterolateral thigh sensory disturbance. As the POFC area, HBL, and anterolateral thigh sensory disturbance were measured postoperatively and may represent concurrent postoperative correlates rather than baseline confounders, they were excluded from the primary model. They were only added to the sensitivity analyses to examine whether the main associations remained materially unchanged. As a post hoc sensitivity analysis of the co-primary outcomes, the primary models were further adjusted for preoperative prothrombin time (PT) and activated partial thromboplastin time (aPTT) to account for potential differences in preoperative coagulation status. Associations of the POFC area with pain and muscle strength were examined using Spearman’s rank correlation coefficients. Post hoc exploratory associations of total blood loss, hidden blood loss, and operative time with pain and muscle strength were also examined using Spearman’s rank correlation coefficients. The WOMAC subscales were analyzed using a mixed model for repeated measures (MMRM) with fixed effects for groups, time point, and group-by-time interaction, adjusted for age, sex, BMI, and the Tönnis classification. Participant identification was included as a random effect, with an unstructured covariance matrix and restricted maximum likelihood estimation. Because group classification was defined by 1-week US findings, WOMAC analyses were intended to describe differences in postoperative recovery trajectories rather than baseline group differences. Time point-specific comparisons were exploratory, and two-sided *p* < 0.05 was considered statistically significant.

## Results

One week postoperatively, 145 hips were classified as non–midline-crossing POFC and 37 as midline-crossing POFC. Baseline characteristics did not differ significantly between the groups (Table 1).

**Table 1:** Preoperative characteristics, operative variables, and postoperative descriptive findings according to the POFC classification

Characteristic	Non–midline-crossing POFC group (*n* = 145 hips)	Midline-crossing POFC group (*n* = 37 hips)	*p*-value
Preoperative characteristics			
Age (years)	67.8 ± 10.1	66.2 ± 9.3	0.45^a^
Sex, n (%)			1.00^b^
Women	123 (84.8%)	32 (86.5%)	
Men	22 (15.2%)	5 (13.5%)	
Height (cm)	155.1 ± 8.1	156.0 ± 7.7	0.61^a^
Weight (kg)	60.3 ± 12.3	62.8 ± 15.9	0.81^a^
Body mass index (kg/m^2^)	24.9 ± 4.0	25.7 ± 5.4	0.64^a^
Tönnis classification system, n (%)			0.42^b^
Grade 0	0 (0%)	0 (0%)	
Grade 1	0 (0%)	0 (0%)	
Grade 2	17 (11.7%)	6 (16.2%)	
Grade 3	128 (88.3%)	31 (83.8%)	
Antiplatelet therapy, n (%)	9 (6.2%)	0 (0%)	0.20^b^
Anticoagulant therapy, n (%)	2 (1.4%)	1 (2.7%)	0.50^b^
Prothrombin time (s), median (IQR)	11.4 (11.0–11.7)	11.4 (11.0–11.6)	0.55^c^
Activated partial thromboplastin time (s), median (IQR)	27.6 (26.2–29.6)	27.5 (25.5–29.5)	0.87^c^
Preoperative knee extension torque (Nm/kg)	0.94 ± 0.34	0.96 ± 0.28	0.42^a^
Operative variables			
Operative time (min)	31.9 ± 7.0	30.6 ± 5.2	0.40^a^
Total blood loss (mL)	596.6 ± 266.3	673.2 ± 251.6	0.06^a^
Hidden blood loss (mL)	390.2 ± 242.8	435.1 ± 212.3	0.13^a^
Postoperative course			
Postoperative length of stay (days), median (IQR)	14 (13–14)	14 (14–14)	0.97^c^
Concurrent postoperative descriptive findings at 1 week			
Sensory disturbance in the anterolateral thigh, n/N (%)	34/145 (23.4%)	11/37 (29.7%)	0.52^b^
Change in thigh circumference (cm)	2.4 ± 1.5	2.7 ± 1.4	0.23^a^

Values are presented as mean ± standard deviation, median (interquartile range), or n (%), as appropriate. For sensory disturbances in the anterolateral thigh, n/N indicates the number of affected cases relative to the total number of cases in each group. The postoperative variables are presented for descriptive purposes and are not baseline variables

*POFC* postoperative fluid collection

^a^Data analyses were performed using Student’s t-test

^b^Data analyses were performed using Fisher’s exact test

^c^Data analyses were performed using the Mann–Whitney U test

Table 2 summarizes reliability results. ICC (1,1) values for the ASIS–proximal neurovascular bundle distance and POFC area were 0.87 and 0.94, with ICC (2,1) values of 0.81 and 0.86, respectively. Agreement for midline-crossing POFC was high, with an intra-rater kappa of 0.90 and inter-rater kappa of 0.81 [44].

**Table 2:** Reliability of US measurements of proximal neurovascular bundle location and POFC area

**Intra-rater reliability (test – retest; ICC [1, 1]/Cohen’s Kappa)**						
	Test 1	Test 2	ICC/Cohen’s kappa	95% CI	MDD95%	Percent agreement
ASIS–proximal neurovascular bundle distance (cm)	12.5 ± 1.9	12.8 ± 1.8	0.87	0.70–0.94	1.9	
POFC area (mm^2^)	331.2 ± 364.5	341.3 ± 375.6	0.94	0.87–0.98	238.1	
Midline-crossing POFC assessment (present/absent)			0.90	0.79–1.00		96.7%
**Interrater reliability (between raters; ICC [2, 1]/Cohen’s Kappa)**						
	Rater 1	Rater 2	ICC/Cohen’s kappa	95% CI	MDD95%	Percent agreement
ASIS–proximal neurovascular bundle distance (cm)	12.7 ± 1.5	12.5 ± 1.7	0.81	0.58–0.92	2.0	
POFC area (mm^2^)	336.2 ± 364.8	396.3 ± 383.2	0.86	0.68–0.94	391.5	
Midline-crossing POFC assessment (present/absent)			0.81	0.67–0.96		94.0%

Values are presented as mean ± standard deviation. Reliability of continuous variables (ASIS–proximal neurovascular bundle distance and POFC area) was assessed using intraclass correlation coefficients (ICCs). Agreement for the categorical assessment of the midline-crossing POFC (present/absent) was assessed using Cohen’s kappa coefficient along with the 95% confidence interval (95% CI) and percentage agreement. MDD95% (minimal detectable difference at the 95% confidence level) was applied only to continuous variables

*Abbreviations: US* ultrasound, *ASIS* anterior superior iliac spine, *ICC* intraclass correlation coefficient, *POFC* postoperative fluid collection, *CI* confidence interval, *MDD95%* minimal detectable difference at the 95% confidence level

Proximal anterolateral thigh pain and knee extension torque were analyzed in 182 and 176 hips, respectively. In ANCOVA adjusted for age, sex, BMI, Tönnis classification, and operative time, the midline-crossing POFC group had greater proximal anterolateral thigh pain during walking at 1 week postoperatively than the non–midline-crossing POFC group (adjusted mean difference, 3.35; 95% CI 2.88–3.81; Holm-adjusted *p* < 0.001). For knee extension torque at 1 week postoperatively, the model was additionally adjusted for preoperative knee extension torque, and the midline-crossing POFC group had lower values than the non–midline-crossing POFC group (adjusted mean difference, − 0.31 Nm/kg; 95% CI: − 0.38 to − 0.24; Holm-adjusted *p* < 0.001). Distal anterolateral thigh pain during walking also differed between the groups (adjusted mean difference, 2.07; 95% CI 1.37–2.77; *p* < 0.001) (Table 3). In sensitivity analyses additionally adjusted for the POFC area, HBL, antithrombotic drug use, and anterolateral thigh sensory disturbance, the results were similar to those of the main analyses, and the adjusted between-group differences remained largely unchanged (proximal NRS, 3.55 points; distal NRS, 2.02 points; and knee extension torque, − 0.32 Nm/kg; all *p* < 0.001) (Supplementary Table 1; Additional file 1). In an additional post hoc sensitivity analysis of the co-primary outcomes, further adjustment of the primary models for preoperative PT and aPTT did not materially alter the associations. The adjusted between-group difference was 3.33 points (95% CI 2.86–3.79; Holm-adjusted *p* < 0.001) for proximal anterolateral thigh pain during walking and − 0.32 Nm/kg (95% CI, − 0.39 to − 0.24; Holm-adjusted *p* < 0.001) for knee extension torque. In a post hoc descriptive overlap analysis among patients with complete data for both co-primary outcomes (*n* = 176), greater walking pain was defined as proximal anterolateral thigh pain during walking in the highest quartile (NRS ≥ 4), and knee weakness was defined as knee extension torque at 1 week in the lowest quartile (≤ 0.611 Nm/kg). Twenty patients had both greater walking pain and knee weakness. Among patients with greater walking pain, 20 of 46 patients (43.5%) also had knee weakness; among patients with knee weakness, 20 of 44 patients (45.5%) also had greater walking pain. These findings indicate that greater walking pain and knee weakness partially overlapped but were not identical clinical findings. In the post hoc descriptive assessment, the median first documented day of walking independence was postoperative day 9 (interquartile range [IQR], 8–11). The median postoperative length of stay was 14 days.

**Table 3:** Adjusted between-group differences in pain and muscle strength

**Outcome**	**Non-midline-crossing POFC group (*n* = 145)**	**Midline-crossing POFC group (*n* = 37)**	**Adjusted mean difference (**midline-crossing – non-midline-crossing**)**	**95% CI**	***p*-value**
Pain during walking at 1 week postoperatively, NRS (0–10), and proximal anterolateral thigh	2.06 ± 1.22	5.43 ± 1.46	3.35	2.88–3.81	< 0.001^a^
Pain during walking at 1 week postoperatively, NRS (0–10), and distal anterolateral thigh	1.40 ± 1.71	3.57 ± 2.01	2.07	1.37–2.77	< 0.001
Knee extension torque at 1 week postoperatively (Nm/kg)	0.88 ± 0.28	0.59 ± 0.19	− 0.31	− 0.38 to − 0.24	< 0.001^a^

Observed values are presented as mean ± standard deviation. Between-group differences are shown as adjusted least squares mean differences estimated from the analysis of covariance models adjusted for age, sex, body mass index, Tönnis classification, and operative time. The knee extension torque at 1 week postoperatively was additionally adjusted for the preoperative knee extension torque

*CI* confidence interval, *NRS* numerical rating scale (0–10), *POFC* postoperative fluid collection

^a^Holm adjustment was applied to co-primary outcomes

The POFC area was weakly correlated with pain in the proximal and distal anterolateral thighs (*r* = 0.31, *p* < 0.001 and *r* = 0.19, *p* = 0.017, respectively) and with knee extension torque at 1 week postoperatively (*r* = − 0.24, *p* < 0.001). In post hoc exploratory analyses, total blood loss, hidden blood loss, and operative time showed no more than weak correlations with pain and knee extension torque. Total blood loss was weakly correlated with distal anterolateral thigh pain during walking (*r* = 0.16, *p* = 0.043) and knee extension torque (*r* = − 0.20, *p* = 0.009), whereas hidden blood loss and operative time were not meaningfully correlated with the clinical outcomes.

**Fig. 5:**
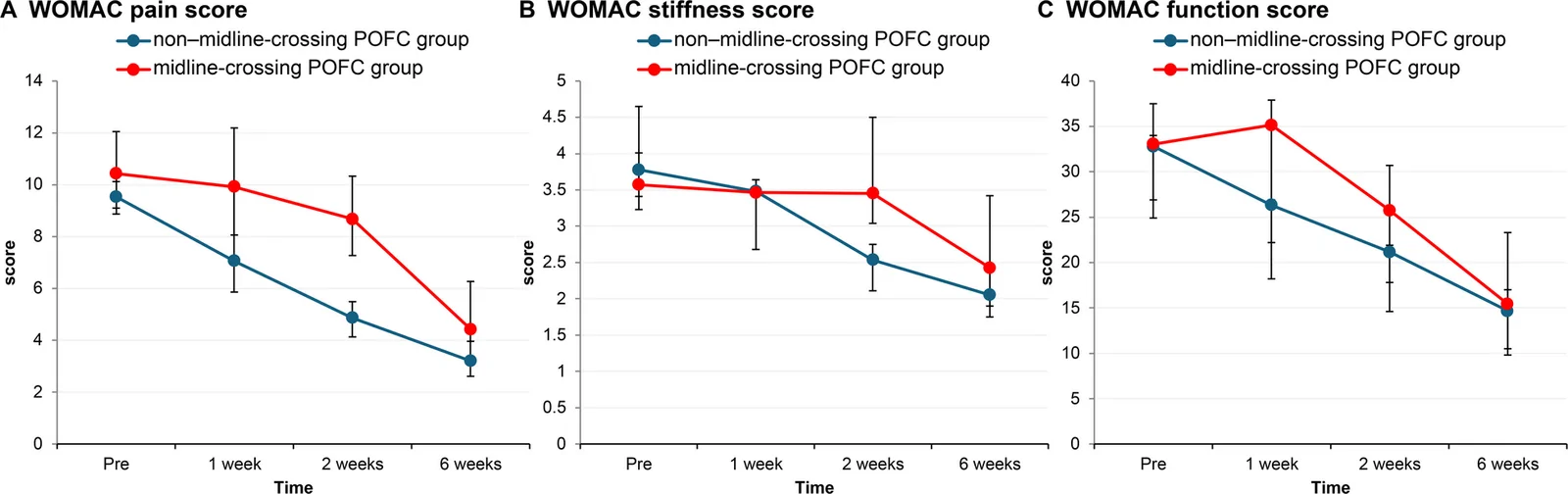
Changes in WOMAC subscale scores at each postoperative time point in the two groups. Error bars indicate 95% confidence intervals (95% CIs). WOMAC, Western Ontario and McMaster Universities Osteoarthritis Index

For WOMAC pain, the MMRM showed a significant group-by-time interaction (*p* < 0.01), with higher values in the midline-crossing POFC group at 1 and 2 weeks but not at 6 weeks. WOMAC stiffness and function showed significant time effects but no significant group effects or group-by-time interactions; therefore, time-point-specific between-group comparisons should be interpreted exploratorily (Fig. 5 and Supplementary Table 2; Additional file 2).

In the longitudinal subset enrolled after 2- and 6-week ultrasound follow-up was added to the protocol (*n* = 57), midline-crossing POFC was observed in 12 of 57 patients (21.1%) at 1 week, 8 of 57 (14.0%) at 2 weeks, and 0 at 6 weeks, with no new or worsening findings during follow-up (Supplementary Table 3; Additional file 3).

## Discussion

In this prospective cohort study, a US-defined midline-crossing POFC at a prespecified reference cross-section (1 week after DAA-THA) was associated with greater proximal anterolateral thigh pain during walking and lower knee extension torque. These associations were observed in the adjusted analyses and remained materially similar in sensitivity analyses. WOMAC pain also showed a significant group-by-time interaction, suggesting that early postoperative recovery may differ according to midline-crossing POFC.

To date, knowledge of the neurovascular structures around the distal DAA interval has been derived mainly from anatomic studies showing potential risks of distal extension and surgical exposure [9, 11, 12]. In contrast, few in vivo studies have evaluated POFC using a simple and reproducible positional classification and examined its association with early postoperative symptoms. In this study, a landmark-based US classification using the femoral midline on short-axis images identified a subgroup of patients with worse early pain and lower knee extensor performance. From a clinical standpoint, these findings suggest that the positional relationship of POFC at the reference cross-section may help interpret disproportionate early postoperative symptoms after DAA-THA.

The midline-crossing POFC group had significantly greater proximal anterolateral thigh pain during walking at 1 week postoperatively. As anterolateral thigh symptoms after DAA-THA are likely multifactorial [45–48], these findings should not be interpreted as evidence of a single mechanism. However, the associations remained materially similar in sensitivity analyses that additionally adjusted for POFC area, HBL, antithrombotic drug use, and anterolateral thigh sensory disturbance.

The knee extension torque was also significantly lower at 1 week postoperatively in the midline-crossing POFC group. Experimental pain studies have shown that pain can reduce quadriceps activation and knee extension torque and attenuate knee extensor moments during walking [7, 8], supporting pain-related inhibition as one plausible contributor to reduced knee extensor performance. However, the extent to which pain itself contributed to the lower knee extension torque observed in the present study cannot be determined. Because the vastus lateralis is innervated by a motor branch of the femoral nerve that courses distal to the DAA interval [10], POFC extending toward the estimated course region may also be associated with weakness through local swelling or changes in the local tissue environment. However, this finding should not be interpreted as direct evidence of neurovascular involvement. The midline-crossing POFC pattern may also reflect greater local tissue disruption, surgical dissection, or postoperative tissue reaction. Ultrasound and electromyography (EMG) provide different types of information and should be considered complementary rather than interchangeable assessments. EMG would be more appropriate for directly evaluating muscle activation, neuromuscular inhibition, or electrophysiologic nerve dysfunction, whereas US can provide anatomical and positional information regarding POFC relative to the estimated course of the proximal neurovascular bundle.

Although the POFC area significantly correlated with pain and knee extension torque, the correlation coefficients were small. Additionally, between-group differences according to the midline-crossing status remained similar after additional adjustment for the POFC area and other postoperative factors. These findings suggest that the overall size of the fluid collection alone does not adequately explain the observed differences. Therefore, the midline-crossing classification may reflect a positional aspect of POFC that is not captured by area alone; however, this interpretation should be considered exploratory.

The clinical relevance of these findings should be interpreted in the context of symptom magnitude. The adjusted between-group difference in proximal anterolateral thigh pain was 3.35 points on a 0–10 NRS, which exceeds previously reported minimal clinically important difference (MCID) values for postoperative pain [49, 50]. In contrast, to our knowledge, no validated MCID has been established for knee extension torque during the early postoperative period after DAA-THA. Therefore, we avoided interpreting the torque difference using an MCID threshold and instead described the magnitude of the observed between-group difference.

One early postoperative THA study showed that knee-extension strength and functional performance were both substantially reduced at discharge, although changes in knee-extension strength were not correlated with changes in functional performance [38]. Accordingly, the adjusted between-group difference in knee extension torque (0.31 Nm/kg lower in the midline-crossing POFC group) may be relevant to early rehabilitation tasks requiring knee extensor control. However, because no validated MCID is available for this measure and the present study did not prospectively evaluate knee extension torque as a predictor of gait recovery, rehabilitation progression, or walking independence, the functional consequences of this magnitude cannot be established from the present data.

Clinical assessment using patient-reported pain and knee extensor strength testing remains the primary method for identifying patients with greater-than-expected early postoperative symptoms. The added value of US is not to replace these assessments, but to provide anatomical and positional information regarding POFC at the reference cross-section that cannot be obtained from symptom severity or strength testing alone. Clinically, routine ultrasound screening of all patients at 1 week after DAA-THA is not recommended based on the present findings. Rather, US evaluation at the reference cross-section may be considered as an adjunctive assessment in selected patients who present approximately 1 week after DAA-THA with proximal anterolateral thigh pain and reduced knee extensor performance that are more pronounced than expected for the usual postoperative course. Detection of midline-crossing POFC would not, by itself, warrant intervention, but may assist in symptom interpretation and short-term follow-up. Whether this ultrasonographic finding should alter rehabilitation progression requires further study. Although midline-crossing POFC was associated with greater early pain and lower knee extension torque, the present observational study does not establish that reducing POFC by postoperative drainage would improve these outcomes. Previous studies, including a systematic review of primary THA and a randomized trial of direct anterior THA, have also found no clear clinical benefit supporting the routine use of closed suction drainage [51, 52]. Because drainage and no-drainage strategies were not compared, the present findings do not support recommending routine postoperative drainage after DAA-THA.

The midline-crossing POFC group showed higher WOMAC pain scores at 1 and 2 weeks, but not at 6 weeks, suggesting that the association was most evident during early postoperative recovery. In the longitudinal subset, the proportion of patients with midline-crossing POFC decreased over time, and no midline-crossing POFC was observed at 6 weeks, with no new or worsening findings during follow-up. These findings suggest that the midline-crossing POFC pattern was predominantly transient within the observed 6-week period. However, because longitudinal ultrasound follow-up was limited to 6 weeks and was available only in a subset of patients, the present study cannot determine whether midline-crossing POFC persists beyond 6 weeks in some patients or whether such persistence has longer-term clinical implications. Longer-term imaging and clinical follow-up are needed to address this issue. Because perioperative analgesia was standardized according to the institutional protocol, analgesic consumption was not analyzed as a separate recovery outcome.

This study has several limitations. This single-center prospective cohort study was conducted by a single experienced surgeon, and the cohort predominantly comprised patients with advanced osteoarthritis (Tönnis grade 3). In addition, the same implant system and standardized surgical and perioperative protocols were used throughout the study. Although these features increased consistency within the cohort, they may limit the generalizability of our findings to patients with less advanced osteoarthritis, other surgeons or institutions, different implant systems, and variations in surgical technique or perioperative management. The group classification and co-primary outcomes were assessed at the same 1-week postoperative time point; therefore, the findings should be interpreted as contemporaneous associations rather than evidence of causality or established prognostic value. Individual-level frequency and cumulative exposure to ice application were not systematically recorded; therefore, the possibility that variation in ice exposure influenced the pain outcomes cannot be completely excluded. The post hoc overlap analysis, walking-independence assessment, and surgical-factor correlation analyses were exploratory and should be interpreted descriptively. POFC was defined using US without aspiration or definitive imaging-based characterization, and a strict differentiation between hematoma and seroma was not possible. An objective electrophysiologic assessment of nerve function or muscle activation, such as EMG, was not performed. Additionally, because the postoperative positional relationship between POFC and the proximal neurovascular bundle was inferred using the femoral midline as a landmark rather than directly demonstrated, the classification should be interpreted as an operational surrogate rather than as direct evidence of bundle contact or compression. Although ultrasonographic measurement of the ASIS–proximal neurovascular bundle distance showed good agreement with magnetic resonance imaging (MRI)-based measurement in healthy adults [53], these findings supported the validity of the ultrasonographic procedure for localizing the proximal neurovascular bundle but did not establish the postoperative spatial relationship between POFC and the bundle. Future studies using MRI or multimodal ultrasonography incorporating Doppler or elastography in postoperative patients may help characterize this relationship more directly and further validate the present positional classification.

## Conclusions

One week after DAA-THA, midline-crossing POFC on a landmark-based reference cross-section was associated with greater walking pain and lower knee extension torque. This finding should be interpreted as an ultrasonographic correlate of more pronounced early symptoms rather than direct evidence of severe neurologic injury or neurovascular compression. In patients whose early proximal anterolateral thigh pain and reduced knee extensor performance appear to be greater than expected after DAA-THA, US assessment at this reference level may provide useful adjunctive information for clinical assessment and short-term follow-up.

## Supplementary Information

Additional file 1. Supplementary Table 1. Adjusted between-group differences in pain and muscle strength (sensitivity analysis).

Additional file 2. Supplementary Table 2. Comparison of WOMAC subscales.

Additional file 3. Supplementary Table 3. Longitudinal changes in midline-crossing POFC.

## Data Availability

The datasets generated and/or analyzed during the current study are not publicly available because they contain potentially sensitive clinical information, but are available from the corresponding author on reasonable request.

## Abbreviations

ANCOVA: Analysis of covariance
aPTT: Activated partial thromboplastin time
ASIS: Anterior superior iliac spine
BMI: Body mass index
CI: Confidence interval
DAA: Direct anterior approach
EMG: Electromyography
HBL: Hidden blood loss
ICC: Intraclass correlation coefficient
IQR: Interquartile range
LFCA: Lateral femoral circumflex artery
MCID: Minimal clinically important difference
MDD95%: Minimal detectable difference at the 95% confidence level
MMRM: Mixed model for repeated measures
MNVL: Motor nerve to the vastus lateralis
MRI: Magnetic resonance imaging
NRS: Numerical rating scale
PENG: Pericapsular nerve group
POFC: Postoperative fluid collection
PT: Prothrombin time
THA: Total hip arthroplasty
US: Ultrasound
WOMAC: Western Ontario and McMaster Universities Osteoarthritis Index
