# Supplementary material for: Postoperative fluid collection at the proximal neurovascular bundle level is associated with early pain and reduced knee extensor strength after direct anterior total hip arthroplasty
Source: Arthroplasty. 2026 Nov 9;8:87. doi: 10.1186/s42836-026-00443-1 (PMC13746599; doi:10.1186/s42836-026-00443-1)
Supplement: Supplementary file 1 [file 42836_2026_443_MOESM1_ESM.docx]

Supplementary Table 1. Adjusted between-group differences in pain and muscle strength (sensitivity analysis)

| Outcome | LS mean difference (**midline-crossing – non–midline-crossing**) | 95% CI | *P*-value |
| --- | --- | --- | --- |
| Pain during walking at 1 week postoperatively, NRS (0–10), and proximal anterolateral thigh | 3.55 | 2.98–4.12 | <0.001 |
| Pain during walking at 1 week postoperatively, NRS (0–10), and distal anterolateral thigh | 2.02 | 1.21–2.83 | <0.001 |
| Knee extension torque at 1 week postoperatively (Nm/kg) | −0.32 | −0.42 to −0.23 | <0.001 |

Adjusted mean values represent estimated marginal means (least squares means) derived from the analysis of covariance models adjusted for age, sex, body mass index, Tönnis classification, operative time, postoperative fluid collection area, hidden blood loss, antithrombotic use (anticoagulants and/or antiplatelet agents), and the presence or absence of sensory disturbance in the anterolateral thigh. The knee extension torque at 1 week postoperatively was additionally adjusted for the preoperative knee extension torque.

Abbreviations: CI, confidence interval; LS mean, least squares mean; NRS, numerical rating scale (0–10)
