# Supplementary material for: Postoperative fluid collection at the proximal neurovascular bundle level is associated with early pain and reduced knee extensor strength after direct anterior total hip arthroplasty
Source: Arthroplasty. 2026 Nov 9;8:87. doi: 10.1186/s42836-026-00443-1 (PMC13746599; doi:10.1186/s42836-026-00443-1)
Supplement: Supplementary file 2 [file 42836_2026_443_MOESM2_ESM.docx]

Supplementary Table 2. Comparison of WOMAC subscales

| Outcomes | Non–midline-crossing POFC group (n=145) | Midline-crossing POFC group (n=37) | LS mean difference | 95% CI | *P*-value |
| --- | --- | --- | --- | --- | --- |
| WOMAC pain score |  |  |  |  |  |
| Preoperative | 9.57 ± 4.15 | 10.43 ± 3.38 | 0.83 | −0.50 to 2.17 | 0.22 |
| 1 week | 7.32 ± 4.30 | 10.13 ± 3.89 | 2.97 | 0.97 to 4.97 | <0.01 |
| 2 weeks | 5.11 ± 3.79 | 8.71 ± 3.61 | 3.56 | 2.17 to 4.95 | <0.01 |
| 6 weeks | 3.33 ± 2.94 | 4.50 ± 2.87 | 1.13 | −0.23 to 2.48 | 0.10 |
| WOMAC stiffness score |  |  |  |  |  |
| Preoperative | 3.82 ± 1.98 | 3.62 ± 1.80 | −0.21 | −0.85 to 0.44 | 0.53 |
| 1 week | 3.61 ± 1.98 | 3.80 ± 2.08 | 0.48 | −0.48 to 1.44 | 0.33 |
| 2 weeks | 2.60 ± 1.77 | 3.38 ± 1.76 | 0.79 | 0.12 to 1.46 | 0.02 |
| 6 weeks | 2.13 ± 1.45 | 2.47 ± 1.42 | 0.33 | −0.32 to 0.99 | 0.32 |
| WOMAC function score |  |  |  |  |  |
| Preoperative | 32.92 ± 13.94 | 33.54 ± 12.02 | 0.62 | −3.89 to 5.13 | 0.79 |
| 1 week | 27.09 ± 13.57 | 35.80 ± 12.58 | 6.82 | 0.48 to 13.16 | 0.04 |
| 2 weeks | 21.75 ± 12.83 | 26.59 ± 11.58 | 4.63 | −0.04 to 9.30 | 0.05 |
| 6 weeks | 14.69 ± 10.49 | 16.92 ± 10.29 | 2.01 | −2.56 to 6.58 | 0.39 |

Values for each time point and group are presented as observed mean ± standard deviation. Between-group differences are shown as least squares mean differences (LS mean differences: midline-crossing POFC group − non–midline-crossing POFC group) estimated from the mixed model for repeated measures, with 95% confidence intervals (95% CIs) and P-values. Fixed effects included group, time point, and group-by-time interaction, with age, sex, BMI, and Tönnis classification as covariates.

WOMAC, Western Ontario and McMaster Universities Osteoarthritis Index; POFC, postoperative fluid collection; LS mean, least squares mean; CI, confidence interval
