# Supplementary material for: Postoperative fluid collection at the proximal neurovascular bundle level is associated with early pain and reduced knee extensor strength after direct anterior total hip arthroplasty
Source: Arthroplasty. 2026 Nov 9;8:87. doi: 10.1186/s42836-026-00443-1 (PMC13746599; doi:10.1186/s42836-026-00443-1)
Supplement: Supplementary file 3 [file 42836_2026_443_MOESM3_ESM.docx]

Supplementary Table 3. Longitudinal changes in midline-crossing POFC

| Assessment time point | Number of evaluated cases (n) | Midline-crossing POFC (%) |
| --- | --- | --- |
| 1 week postoperatively | 57 | 12 (21.1%) |
| 2 weeks postoperatively | 57 | 8 (14%) |
| 6 weeks postoperatively | 57 | 0 (0%) |

Midline-crossing POFC was assessed using the same ultrasound evaluation criteria as in the primary analysis. No patient showed a newly developed midline-crossing POFC or worsening of findings during follow-up. This analysis was exploratory, and no statistical tests were performed.

POFC, postoperative fluid collection
